# Retinal Sensitivity in *KCNV2*-Associated Retinopathy

**DOI:** 10.1167/iovs.66.1.26

**Published:** 2025-01-10

**Authors:** Thales A. C. de Guimaraes, Isabela M. C. de Guimaraes, Manickam Nick Muthiah, Angelos Kalitzeos, Michel Michaelides

**Affiliations:** 1 UCL Institute of Ophthalmology, University College London, London, United Kingdom; 2 Moorfields Eye Hospital NHS Foundation Trust, London, United Kingdom; 3 Universidade Sao Leopoldo Mandic, Campinas, Sao Paulo, Brazil; 4 Barts Health NHS Trust, London, United Kingdom

**Keywords:** *KCNV2*, cone-rod dystrophy, microperimetry, photopic, scotopic, inherited retinal dystrophy (IRD)

## Abstract

**Purpose:**

The purpose of this study was to analyze the retinal sensitivity under photopic, mesopic, and scotopic conditions in a cohort of patients affected with *KCNV2*-associated retinopathy.

**Methods:**

Cross-sectional evaluation of molecularly confirmed individuals was conducted. Data were obtained prospectively. The main microperimetry parameters analyzed were mean sensitivity (MS) and number of floor effects (noF) in the central macular zone (CMZ; central 6 degrees) and peripheral macular zone (PMZ; 6 to 10 degrees). Interocular symmetry was also assessed.

**Results:**

The MS under photopic, mesopic, and scotopic conditions were, respectively, 20.2 (6.3–29.5, ±6.5), 16.2 (3.7–23.1, ±5.3), and 1.3 (0–13.3, ±3.8) in the right eyes, and 21.1 (9.8–28.9, ±6.1), 16.8 (9.6–23.5, ±4.0), and 1.1 (0–12.4, ±3.5) in the left eyes. MS was highly symmetric between eyes (Pearson correlation coefficient) in photopic (*r* = 0.96, *P* < 0.0001), mesopic (*r* = 0.9, *P* < 0.0001), and scotopic (*r* = 0.98, *P* < 0.0001) testing conditions. MS was also highly symmetric (Pearson coefficient) in the photopic CMZ (*r* = 0.97, *P* = 0.0001) and PMZ (*r* = 0.95, *P* = 0.0002), mesopic CMZ (*r* = 0.94, *P* = 0.0003), and PMZ (*r* = 0.92, *P* = 0.0009), and scotopic CMZ (*r* = 0.99, *P* < 0.0001) and PMZ (*r* = 0.99, *P* < 0.0001).The mean noF (± standard deviation [SD]; range) was 4.6 (SD = ±5.3, range = 0–16) in photopic, 5.0 (SD = ±5.4, range = 0–15) in mesopic, and 23.2 (SD = ±8.6, range = 0–28) in scotopic conditions. Subject 01-041 was the only affected individual in which no floor effect was found. Simple linear regression revealed a significant inverse relationship between age and MS, and a direct relationship between age and noF.

**Conclusions:**

*KCNV2*-associated retinopathy is a largely symmetric disease from a functional perspective. Despite the early decrease in scotopic retinal sensitivity, our data suggests a large window for photoreceptor rescue with novel treatments, such as gene therapy.

*K* *CNV2*-associated retinopathy (cone dystrophy with supernormal rod responses; OMIM #610356) is an autosomal recessive (AR) form of inherited retinal disease (IRD) with pathognomonic electroretinogram (ERG) waveforms that have been well described in the literature.^1^^–^^4^ This condition was first described by Gouras and collaborators in 1983, and, given the ERG findings, was named cone dystrophy with nyctalopia and supernormal rod responses.^5^

The largest multicentric cohort of affected individuals (*n* = 117) was reported in the *KCNV2* Study Group. The first report established an early disease-onset and outlined ERG changes that were consistent with a stable retinal dysfunction across many decades, as well as increasing the number of novel pathogenic variants; whereas the second report characterized the retinal architecture and structural changes using optical coherence tomography (OCT) and fundus autofluorescence (FAF), which has also been reported in detail by several previous research groups.^6^^–^^11^ Both reports identified a slowly progressive disease – which is rather stable in adulthood after an early decrease in visual acuity – and a large window for therapeutic intervention. The third report then assessed genotype-phenotype correlations and found a relatively greater structural integrity and better BCVA in patients with non-null variants.^12^

There is, however, a lack of data relating to retinal sensitivity, which is crucial for clinical trial design given that mean sensitivity is frequently listed as a primary or secondary outcome in many studies. The purpose of this report is to expand upon previous descriptions by providing detailed phenotypic characteristics of *KCNV2*-associated retinopathy with microperimetry performed under three different conditions – namely photopic, mesopic, and scotopic.

## Methodology

The study protocol adhered to the tenets of the Declaration of Helsinki and received approval from all local ethics committees of the participating institutions. Informed consent was obtained from all adult subjects, whereas informed consent and assent were obtained from parents and children, respectively.

### Patient Identification

Patients were recruited from an ethically approved natural history study in a single center (Moorfields Eye Hospital, London, UK). All patients had molecularly confirmed variants in *KCNV2* and characteristic clinical phenotype. The cohort comprises 24 eyes of 12 affected individuals, and 4 healthy controls.

### Visual Acuity

Best-corrected visual acuity (BCVA) was measured on the same day as part of the protocol of the aforementioned natural history study. BCVA was converted to LogMAR for analysis.^13^

### Microperimetry

All patients had microperimetry testing using the MP3-S (Nidek Inc., Gamagori, Japan). This is the only commercially available fundus-guided perimetry device that allows accurate determination of retinal sensitivity under three different conditions, namely (i) photopic, in which the cones contribute to most of the response, (ii) mesopic, whereas both population of cells (cones and rods) are active and the response to the stimuli is mixed, and (iii) scotopic, in which rod response is isolated after a period of dark adaptation.

**Figure 1.**
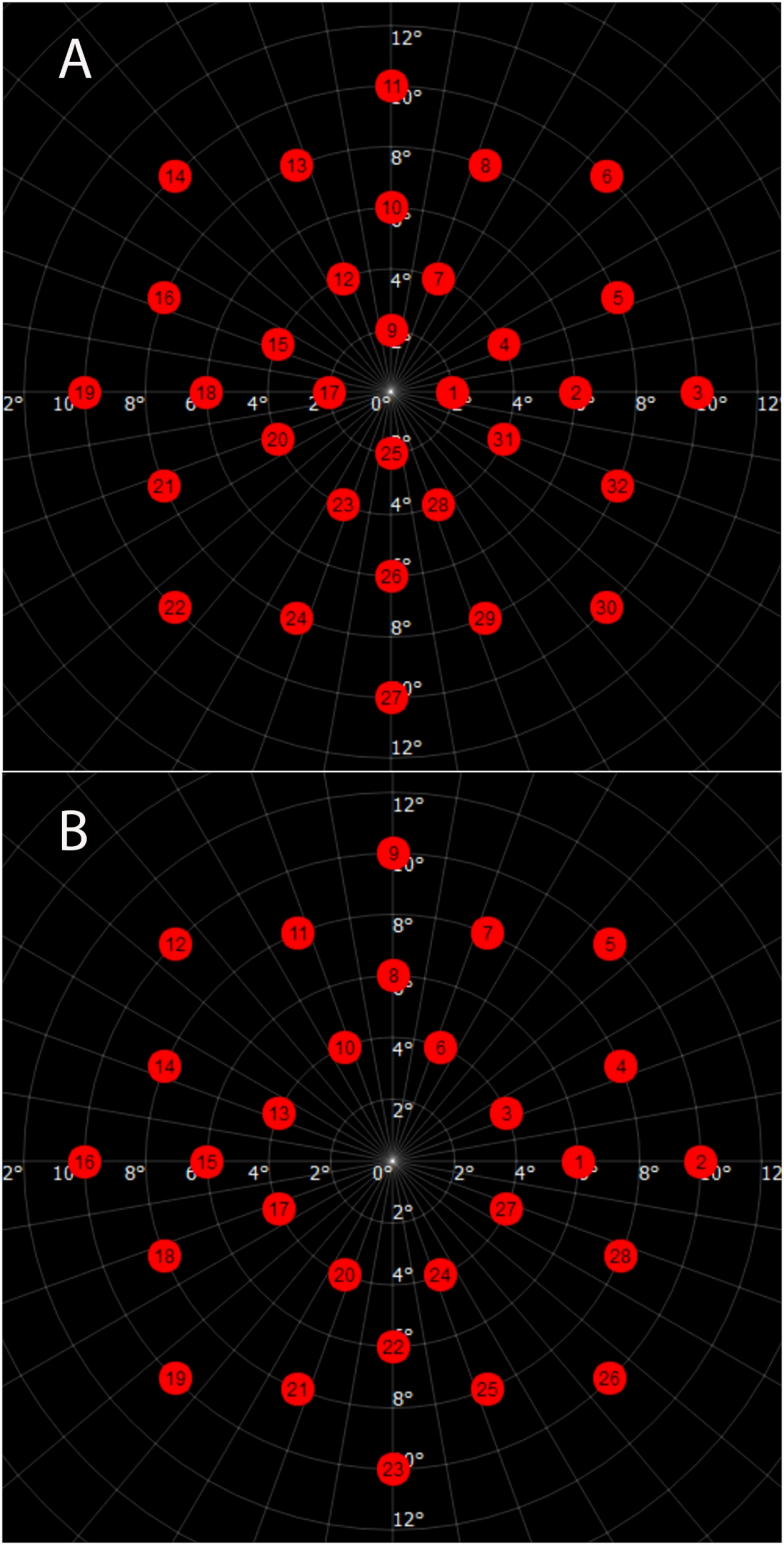
Test grids used for photopic/mesopic (**A**) and scotopic (**B**).

The test was performed without pupil dilation in a completely darkened room. Dark adaptation was only required for the scotopic configuration, in which the patient was kept in the room with a blindfold for 30 minutes prior to the test. The order of the eye tested was always the right eye first, followed by the left eye starting with photopic, then mesopic, and, finally, scotopic. The test consisted of 2 custom-designed radial grids: a 32-point grid to be used for photopic and mesopic, and another 28-point grid to be used in scotopic testing (Fig. 1). Both grids test the central 10 degrees of the macula (10-2). A Goldmann size III stimulus with 200 ms duration was used in a 4-2 strategy, on a background luminance of 31.4 asb (9.99 cd/m²) for photopic, and 4 asb (1.27 cd/m²) for mesopic. The fixation target used is a 1-degree size red cross. The dynamic range of the device in these modalities is 34 decibel (dB). The grid used for scotopic testing is identical to the mesopic/photopic minus the 4 positions within 2 degrees of the fovea (rod-free zone). A Goldmann V stimulus with 200 ms duration in a 4-2 strategy, and a background luminance of 0.003 asb (0.0009 cd/m²) were used. The fixation target is a white circle with a 3-degree radius. If the subject could not see the circle, the examiner changed the target to four crosses, and, if still not visible, the patient was guided verbally based on the fundus image on the screen. The dynamic range of the device in the scotopic setting is 24 dB. The reliability factor threshold was set to 20%, with the test being repeated if necessary. This methodology was fully described and validated in detail in another study.^14^

### Main Outcome Measurements

The main outcomes were mean sensitivity (MS) and the number of floor effects (noF) across all modalities. The mesopic MS was compared in the central macular zone (CMZ; central 6 degrees) and peripheral macular zone (PMZ; 6 to 10 degrees) as the average of both eyes in the healthy controls and the affected individuals. To evaluate trends, age was correlated with MS, noF in each test modality (representing an absolute scotoma, i.e. inability to detect the highest stimuli in any position across the grid), and by assessing the interocular symmetry.

### Statistical Methods

Statistical analysis was performed with the aid of GraphPad Prism 9 (GraphPad Software, San Diego, CA, USA). Parametric and non-parametric tests were used, as well as correlation parameters (either Pearson or Spearman). Significance of all statistical tests was set at *P* < 0.05 and D'Agostino-Pearson test (omnibus K2) was used to determine normality for all variables.

## Results

### Patient Identification and Visual Acuity

**Figure 2.**
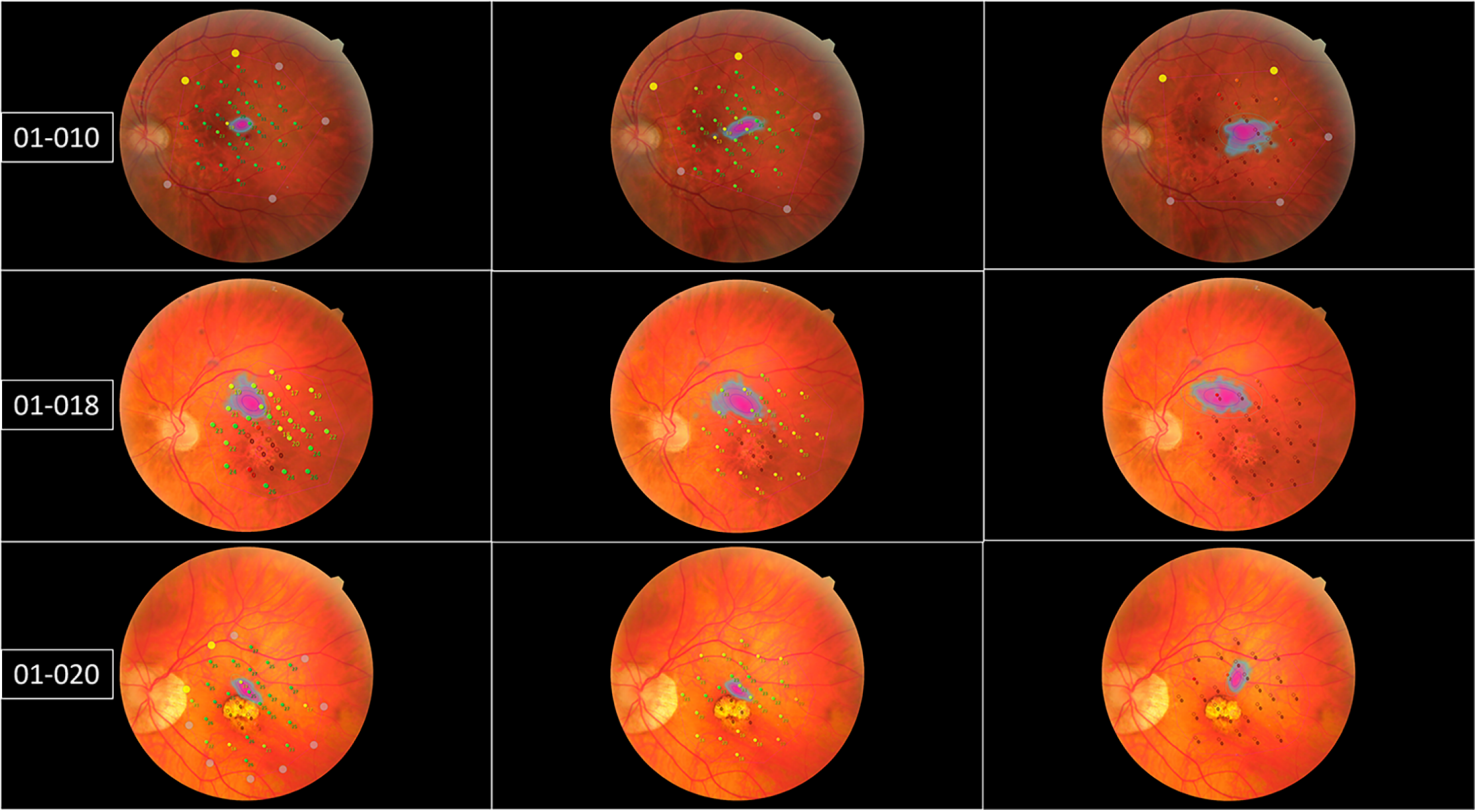
MP3-S printouts of subjects 01-010, 010-018, and 01-020. Each row represents the photopic, mesopic, and scotopic modalities presented, respectively, in sequence from *left* to *right*. Patients performed poorly in scotopic conditions, with the vast majority of test points having a floor effect.

Twenty-four eyes of 12 affected patients (11 unrelated families) and 4 eyes of 2 controls were tested in the full 3-hour long protocol for photopic, mesopic, and scotopic microperimetry. The mean age of the affected individuals and healthy controls was, in years (range; ±SD), 30.5 (range = 13–57, SD = ±14.8) and 31.0 (range = 25–36, SD = ±5.7), respectively. Table 1 summarizes the patients’ molecular genetics, refraction, and the axial length. Figure 2 provides a montage with a few selected examples.

**Table 1.** Molecular Variants, Age When Tested, Spherical Equivalent (sph eq) and Axial Length of All Individuals In This Cohort

ID	Allele 1	Allele 2	Age, Y	SPH EQ OD/OS, D	Axial Length OD/OS, mm	Visual Acuity OD/OS
01-004	c.1381G>T; p.(Gly461*)	c.442G>T; p.(Glu148*)	33	+3.75/+4.00	22.23/21.88	1.0/1.2
01-010	c.778A>T; p.(Lys260*)	c.778A>T; p.(Lys260*)	28	−10.25/−9.50	26.87/26.9	1.1/1.0
01-013	c.224_230delins204	c.224_230delins204	11	−7.00/−7.25	26.43/26.57	0.5/0.6
01-016	c.1381G>T; p.(Gly461Ter)	c.433G>T; p.(Gln145Ter)	49	−1.75/−2.00	23.61/24.41	1.1/1.1
01-017	c.1199delT; p.(Phe400Serfs*54)	c.417C>A; p.(Cys139*)	13	−1.00/−0.50	23.84/23.63	0.9/0.9
01-018	c.1381G>T; p.(Gly461Ter)	c.433G>T; p.(Gln145Ter)	47	−0.50/−2.50	23.57/22.91	1.1/1.1
01-020	c.1199delT; p.(Phe400Serfs*53)	c.8_11delAACA; p.(Lys3fs*9)	54	−10.50/−8.00	28.01/26.57	1.1/1.2
01-021	c.782C>A; p.(Ala261Asp)	c.782C>A; p.(Ala261Asp)	33	+1.50/+0.25	24.42/24.64	1.1/1.1
01-039	c.427G>T; p.(Glu143*)	c.427G>T; p.(Glu143*)	22	−2.75/−2.75	23.91/23.92	0.7/0.6
01-041	c.614_617dupAGCG; p.(207Alafs*166)	c.854T>G; p.(Met285Arg)	17	−1.75/−1.75	24.91/24.67	0.6/0.7
01-043	c.562T>A; p.(Trp188Arg)	c.8_11delAACA; p.(Lys3Argfs*96)	25	−1.75/−3.00	24.14/24.33	1.2/1.1
01-053	c.529T>C; p.(Cys177Arg)	c.755G>A; p.(Ala259Thr)	28	−0.25/−0.25	22.28/22.44	0.5/0.9

D, diopters; mm, millimeters; OD, right eye; OS, left eye.

Visual acuity is expressed in LogMAR.

The mean visual acuity (range; ±SD) was 0.9 LogMAR (range = 0.5–1.2, SD = ±0.3) for the right eyes and 0.95 LogMAR (range = 0.6–1.2, SD = ±0.2) for the left eyes, which was not significantly different (*P* = 0.25, *t* = 1.2, paired *t*-test).

### Microperimetry

The mean photopic, mesopic, and scotopic MS in dB (range; ±SD) in affected individuals was, respectively, 20.2 (range = 6.3–29.5, SD = ±6.5), 16.2 (range = 3.7–23.1, SD = ±5.3), and 1.3 (range = 0–13.3, SD = ±3.8) in the right eyes, and 21.1 (range = 9.8–28.9, SD = ±6.1), 16.8 (range = 9.6–23.5, SD = ±4.0), and 1.1 (range = 0–12.4, SD = ±3.5) in the left eyes; all were significantly different to the controls (*P* < 0.0001 in both eyes; unpaired *t*-test). The corresponding values in the control right eyes were 31.5 (range = 30.9–32.1, SD = ±1.7), 27.8 (range = 27.8–27.8, SD = ±0.2), and 16.7 dB (range = 15.7–17.8, SD = ±1.4). There was a high interocular correlation (Pearson correlation coefficient) in photopic (*r* = 0.96, *P* < 0.0001), mesopic (*r* = 0.9, *P* < 0.0001), and scotopic (*r* = 0.98, *P* < 0.0001).

**Figure 3.**
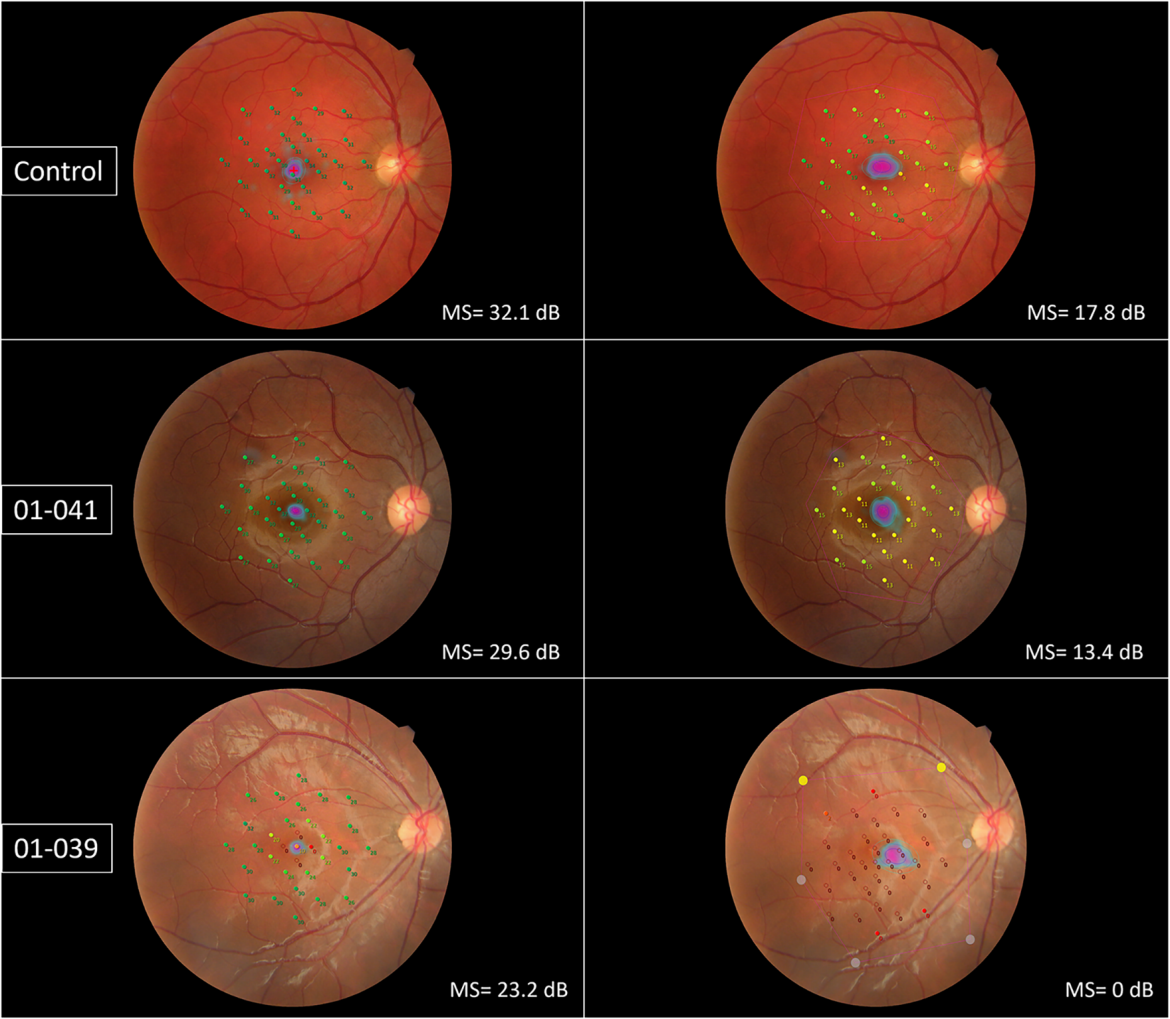
Photopic (*first column*) and scotopic (*second column*) testing of a healthy control (*first row*) and subjects 01-041 (*second row*) and 01-039 (*third row*), with each corresponding mean sensitivity (MS) in the *lower right corner*. The patient identified with the M285R hypomorphic allele (01-041) has a preserved MS, even when compared to one of the mildest phenotypes in the cohort, patient 01-039 (age = 22 years). Of note, he did not have floor effects (i.e. points in which the sensitivity is less than 0 dB).

An outlier was identified in a ROUT test (Q < 1%), subject 01–041, who has the hypomorphic allele M285R. He was not excluded from this analysis as we provided sufficient evidence in a separate study that this represents part of the *KCNV2* disease spectrum.^15^ The MS in the right and left eyes were, respectively, 29.6 and 28.9 dB in photopic, 23.2 and 22.2 dB in mesopic, and 13.4 and 12.4 dB in scotopic testing. This was nominally much higher than the average in affected individuals and comparable to the healthy controls, particularly under photopic conditions (Fig. 3). Similarly, he was the patient that had the highest MS in the scotopic testing and the only affected individual with no floor effects in any position in the grid.

Mesopic microperimetry in the CMZ versus PMZ were then analyzed. In the healthy controls, the mean MS (±SD; range) was 28.1 dB (SD = ±2, range = 25–34) and 27.6 dB (SD = ±2, range = 23–32) in the CMZ and PMZ, respectively. In affected individuals, the mean MS was 13.4 dB (range = 0–25, SD = ±9.2) in the CMZ and 18.1 (range = 0–3, SD = ±11.1) in the PMZ, which was statistically different to the healthy controls (*P* < 0.0001; unpaired *t*-test).

**Figure 4.**
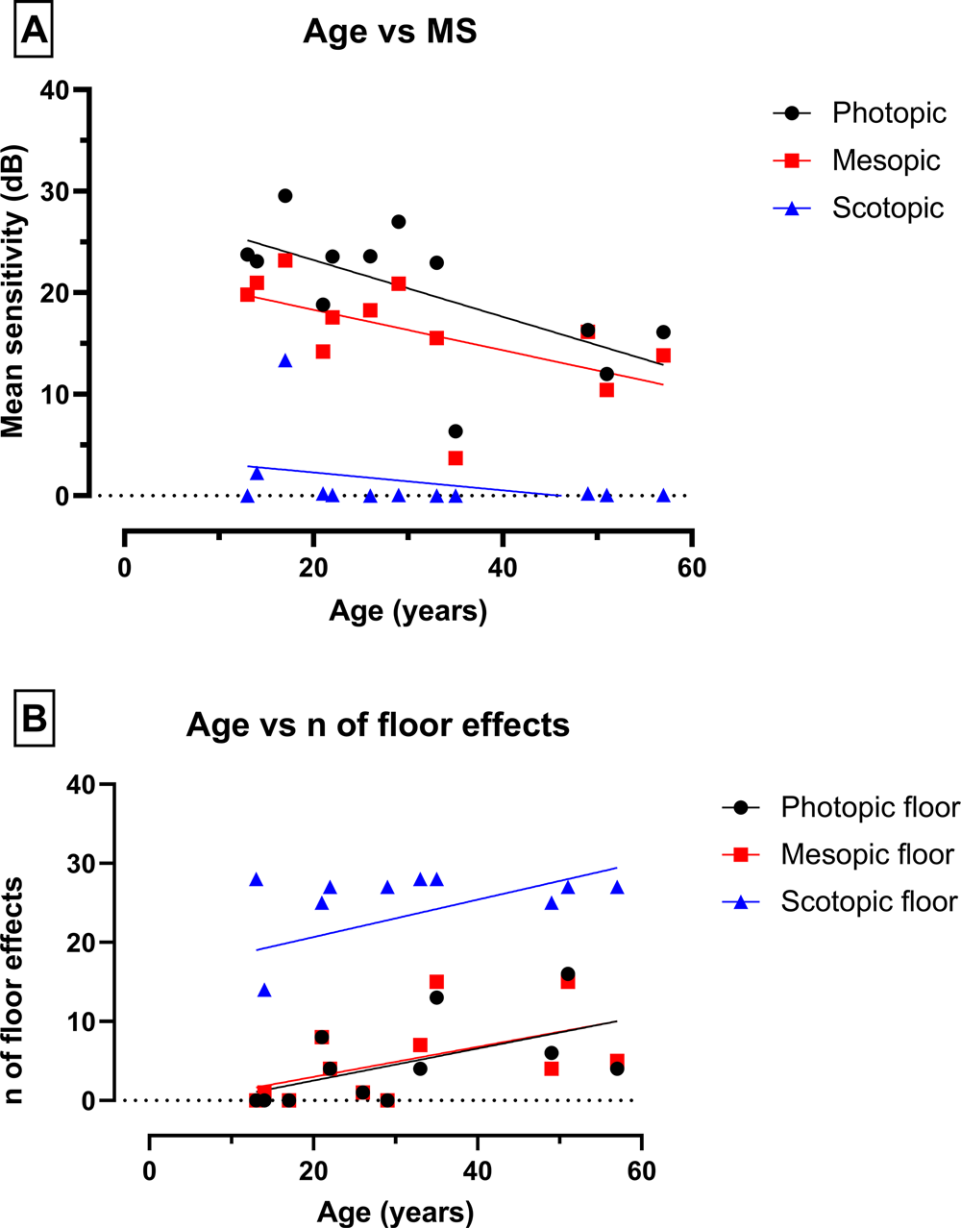
Simple linear regression of (**A**) age versus mean sensitivity (MS), and (**B**) age versus the number of floor effects (noF) under the three different conditions analyzed.

The noFs was also investigated. Similarly, given the high correlation, only values for the right eyes are subsequently described. The mean noF (±SD; range) was 4.6 (SD = ±5.3, range = 0–16) in photopic, 5 (SD = ±5.4, range = 0–15) in mesopic, and 23.2 (SD = ±8.6, range = 0–28) in scotopic conditions. Subject 01-041 was the only affected individual in which no floor effect was found. A simple linear regression model revealed an inverse relationship between age and MS, and a direct relationship between age and noF (Fig. 4). A summary of the microperimetry data described herein is found in Table 2.

**Table 2.** Summary of the Microperimetry Data Under Photopic, Mesopic, and Scotopic Conditions in Both Affected Individuals and Healthy Controls

Mean (Range; ±SD)	Affected Individuals	Controls	*P* Value
Participants	12	4	
Age, y	30.5 (14–57; ±15.5)	31 (25–36; ±5.6)	–
Photopic, dB	20.1 (6.3–29.6; ±7.3)	31.5 (30.9–32.1; ±1.7)	*P* < 0.0001
Mesopic, dB	16.3 (3.7–23.2; ±6.1)	27.8 (27.8–27.8; ±0.2)	*P* < 0.0001
Scotopic, dB	2.0 (0–13.36; ±4.6)	16.7 (15.7–17.8; ±1.5)	*P* < 0.0001
Central macular zone, dB	13.4 (0–25; ±9.2)	28.1 (25–34; ±2)	*P* < 0.0001
Peripheral macular zone, dB	18.1 (0–26; ±7.2)	27.6 (23–32; ±2)	*P* < 0.0001
Floor effects (range, ±SD)			
Photopic, noF	4.4 (0–13; ±4.6)	–	–
Mesopic, noF	4.6 (0–15; ±5.0)	–	–
Scotopic, noF	21.6 (0–28; ±9.8)	–	–

The right eyes are represented herein for each modality, whereas the central macular zone (CMZ) and peripheral macular zone (PMZ) are the average of both eyes under mesopic conditions. The dynamic range of the MP3-S device is 24 dB. Of note, no floor effects were found in the healthy controls. The *P* value here is associated with the unpaired *t*-test.

dB, decibels; noF, number of floor effects.

## Discussion

We provided the first comprehensive evaluation of retinal sensitivity in affected individuals with *KCNV2*-retinopathy following a standardized protocol under different test conditions. This methodology is highly reproducible and has been described in detail in a recently published manuscript.^14^

Our data suggest a possible disease model from a psychophysical perspective. There is a severe reduction of MS under scotopic conditions, with most patients not identifying the brightest stimuli. This is in keeping with the findings from large ERG studies that report almost absent rod responses under scotopic conditions, with a disproportionate improvement with gradually increasing light levels.^3^^,^^4^^,^^16^ It would not be unfeasible to postulate that treatments targeting the photoreceptors could re-establish retinal function under scotopic conditions. Hence, it would be crucial that scotopic microperimetry is used as an outcome measure for future clinical trials, particularly the ones aimed at photoreceptor-directed rescue such as gene therapy.

Subject 01-041 had a considerably higher retinal sensitivity than the rest of the cohort; moreover, he did not display floor effects under any testing condition. This highlights the preserved function in both the cones and rod systems, albeit the clear clinical and molecular diagnosis of the disease, providing more evidence towards the re-classification of the M285R variant. This mutation was previously classified as benign, according to the American College of Medical Genetics (ACMG) criteria, mainly based on its commonality in the African population.^17^ We have provided detailed evidence elsewhere that this represents, in fact, a hypomorphic variant.^15^ First, (i) all in silico tools used, without exception, suggest that this variant is indeed pathogenic, (ii) it represents a highly conserved segment with almost full conservation in Multiz Alignment (100 vertebrates), and (iii) a previous paper has performed functional assays for this mutation, showing slower activation kinetics with a shift in the voltage-dependence of activation to a more depolarized state, demonstrating a reduced activity of the protein.^18^ Furthermore, our patient has pathognomonic clinical findings, including the presence of the well-characterized ERG responses.^3^

Photopic and mesopic microperimetry appear to be relatively preserved throughout the cohort, except in a few cases, even in the presence of floor effects in the center correlating with areas of severe structural damage. The difference in MS in both macular zones, CMZ and PMZ, also appear to directly reflect the underlying structural damage. An interesting hypothesis proposed by Stockman et al. (2014) in their detailed psychophysical study is that, upon measuring the rod critical fusion frequencies, the degree of night blindness was intrinsically dependent on the lighting conditions.^19^ Their data showed that below the –2 log_10_ scotopic luminance range of rod critical fusion frequency, all subjects (*n* = 5) had night blindness, with some level of rod response being measured above that level. This corroborates our findings herein of an extremely reduced – and sometimes absent – retinal sensitivity in scotopic testing with an unexpected increase under mesopic conditions that appears arguably disproportional to the change in background luminance in the device.

When put into context with other variables, age significantly correlated negatively with MS and positively with noF – whereas MS had an inverse relationship with noF. These highlight the progressive nature of the disease, revealing an overall decrease in retinal sensitivity with age and an increase in the number of loci in the retina with no measurable sensitivity. It is also worth noting that functional changes far precede visible structural changes in *KCNV2*-retinopathy given the universally early visual acuity loss characteristic of the disease.^16^ Granted, imaging techniques that provide a high-resolution view of the photoreceptor mosaic, such as adaptive optics scanning light ophthalmoscopy (AOSLO), may provide visualization of these early microscopic changes.

The subjects in this study have the characteristic clinical features of previous data reported in the literature, including a universally decreased BCVA, which limits further attempts at correlation with microperimetry parameters, further highlighting the importance of the latter. In IRDs affecting the central retina, BCVA will undoubtedly be decreased due to the central cone dysfunction, with microperimetry providing measurable retinal sensitivity values surrounding the area of photoreceptor degeneration; thus, allowing for a more comprehensive characterization of retinal function. This has direct implications for patient prognostication, follow-up, and, ultimately, clinical trial eligibility and outcomes.

There are, however, clear limitations to this study, particularly given the cross-sectional nature of the assessments. Data acquisition is challenging given the presence of nystagmus in most subjects tested, although the MP-3S performed well even under these circumstances. We minimized this potential difficulty by selecting subjects that had reasonable quality OCT volume scans that thereby required good fixation. Similarly, the MP3-S is a relatively new device with no large control datasets at present. Furthermore, although the dynamic range is indeed wide for photopic and mesopic testing – and comparable to other commercially available devices^20^ – the authors cannot rule out the floor effects being caused by the smaller range of 24 dB for scotopic testing. We have also not analyzed the preferred retinal locus (PRL) position, which is planned to be published as part of our longitudinal natural history study that is currently ongoing. This may provide further insights into this disease, particularly when correlated to other functional and structural parameters. The authors have not determined the test-retest variability of this method, but the tests had reliability factors well below the preset threshold of 20%; thus, repeated testing was not necessary. Last, although our microperimetry unequivocally shows a trend with age, the authors have not conducted a multi-variant analysis to adjust for possible confounders of age, which represents another limitation.

## Conclusions

In summary, our data suggest that *KCNV2*-retinopathy (i) is a highly symmetric disease from a functional perspective, and that (ii) there is reduction in retinal sensitivity at the central macula, with more preserved peripheral macular sensitivity. Furthermore, rod-mediated retinal sensitivity decreases at very early stages of the disease, possibly at a similar rate to cone-mediated light adapted conditions; although longitudinal studies would be needed to explore this hypothesis. This is supported by a severely decreased MS under scotopic conditions, followed by a decrease in both mesopic and photopic conditions – with these testing conditions possibly reflecting the underlying structural disease pattern. Due to the relatively large window of preserved retinal function, despite an early decrease in retinal sensitivity, *KCNV2*-associated retinopathy is an attractive target for gene therapy.
